# Supplementary material for: T cell receptor–dependent S-acylation of ZAP-70 controls activation of T cells
Source: J Biol Chem. 2021 Jan 19;296:100311. doi: 10.1016/j.jbc.2021.100311 (PMC7949058; doi:10.1016/j.jbc.2021.100311)
Supplement: Figures S1–S6 [file mmc1.docx]

**Supplementary figures**

**
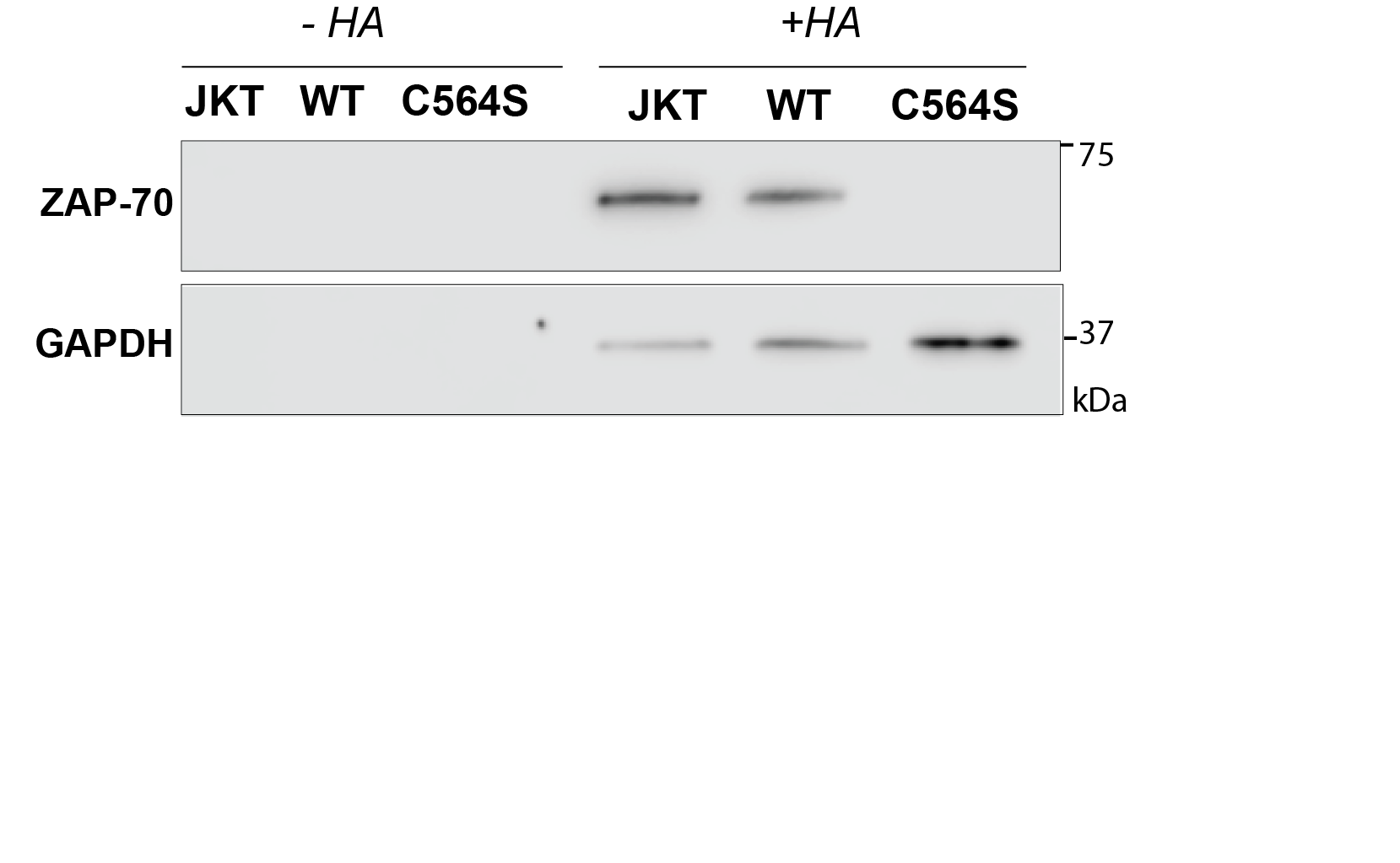
**

**Fig S1. ZAP-70 is S-acylated at cysteine 564.** Identification of ZAP-70 S-acylation site. ABE was performed on Jurkat (JKT) cells or P116 (ZAP-70 -/-) Jurkat T cells stably expressing WT or mutant versions of ZAP-70. Loss of ZAP-70 S-acylation was observed in cells expressing ZAP-70 with C564S mutation compared to Jurkat and WT cells. S-acylation of GAPDH, a known S-acylated protein, served as a positive and loading control.


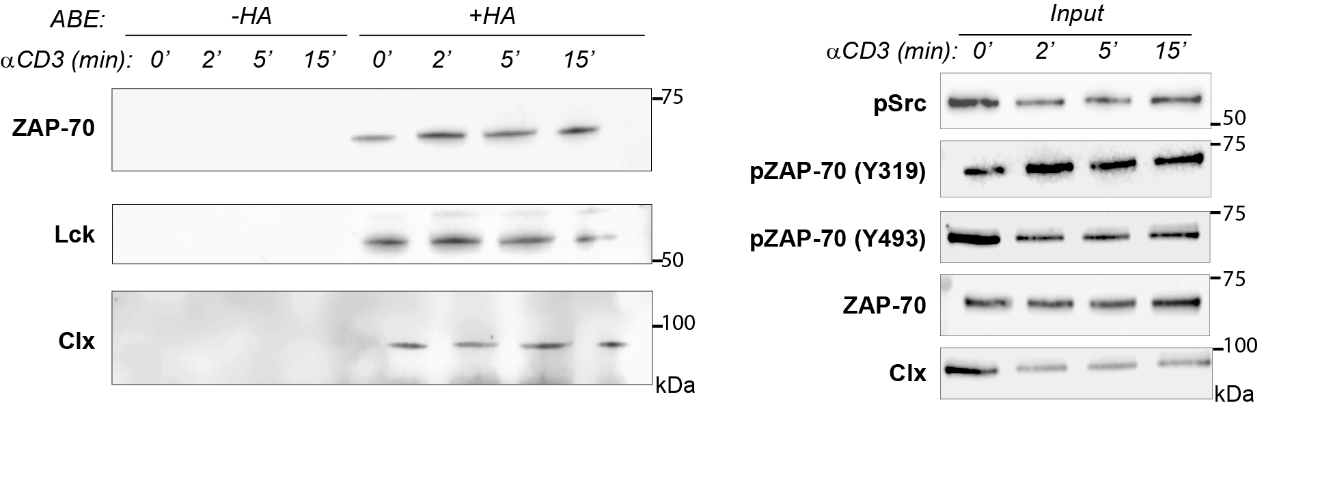


**Fig. S2. Calcium is required for TCR-induced S-acylation of ZAP-70.** Kinetics of agonist-induced S-acylation assessed in J.gamma1 (PLC-γ1 null) Jurkat T cells. Cells were stimulated with 10 µg/ml anti-CD3 antibody for the indicated times and lysates were subject to ABE analysis. Calnexin (Clx), a known S-acylated protein was used as a positive and loading control. Input samples were used to confirm phosphorylation of T cell signaling proteins in response to T cell stimulation. Data shown are representative of 3 independent biological repeats.


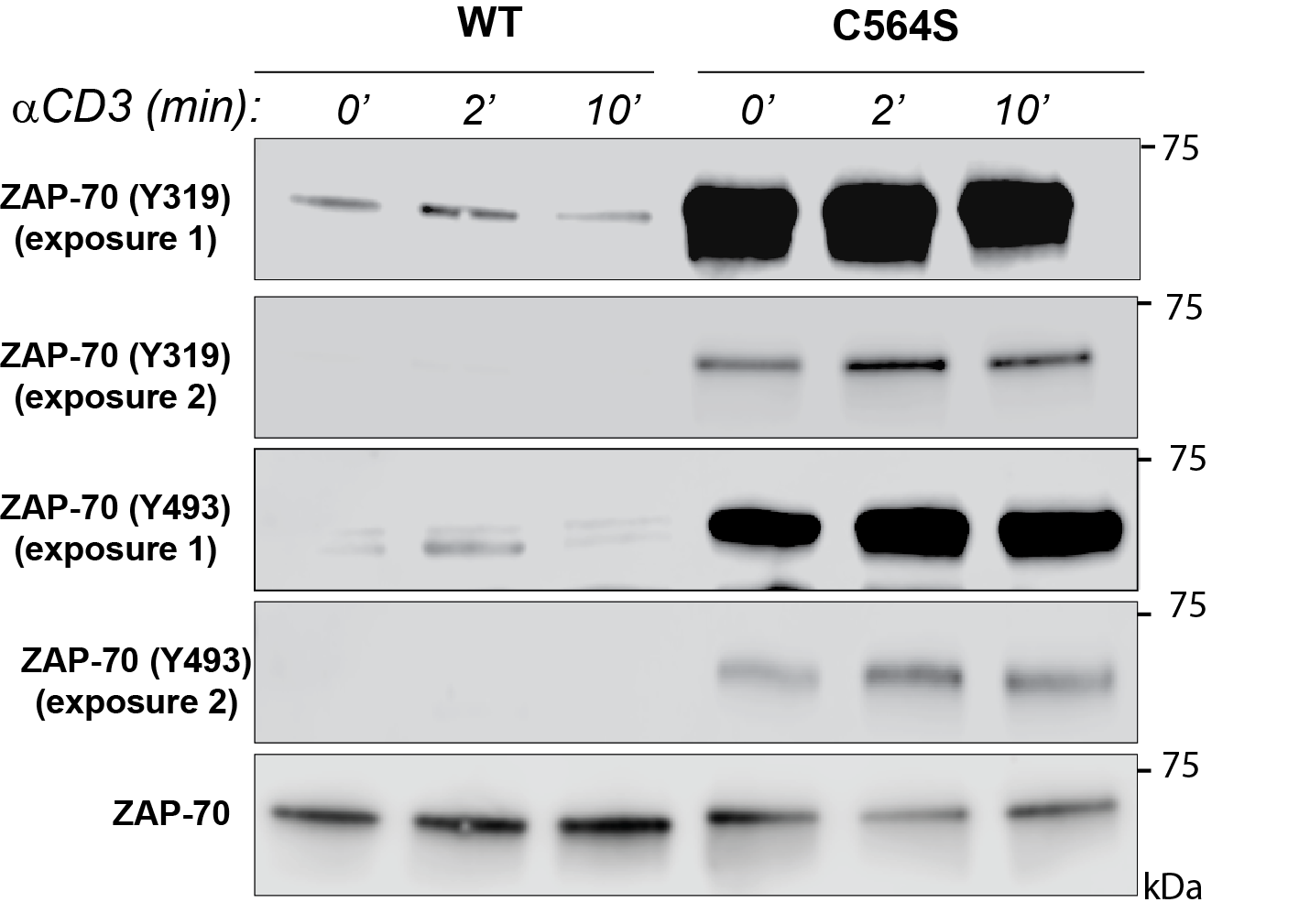


**Fig. S3. Acylation-deficient ZAP-70 exhibits increased phosphorylation at Y319 and Y493.** Western blot analysis of ZAP-70 phosphorylation at Y319 and Y493**.** P116 (ZAP-70 -/-) Jurkat T cells stably expressing WT ZAP-70 or C564S ZAP-70 were stimulated with anti-CD3 antibody for the indicated time points and phosphorylation of ZAP-70 was analyzed by immunoblotting.

**
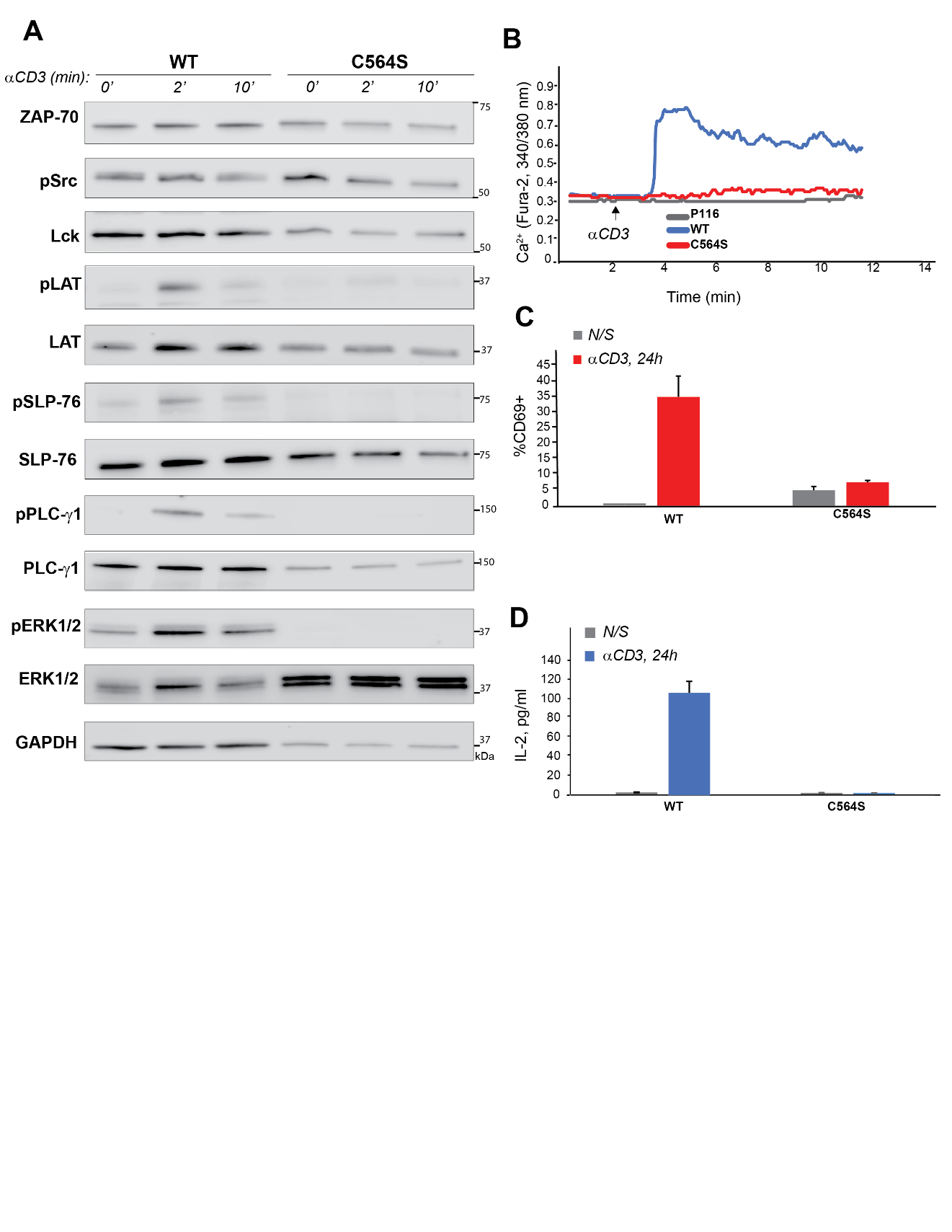
**

**Fig S4. S-acylation of ZAP-70 is required for proximal TCR signaling and T cell activation. (A)** Phosphorylation of proximal TCR signaling proteins in P116 (ZAP-70 -/-) Jurkat T cells stably rescued with WT or acylation-deficient C564S ZAP-70. 1 X 10^7^ cells were stimulated with anti-CD3 antibody for the indicated time point and phosphorylation of TCR signaling proteins was assayed by immunoblotting. Total protein levels shown as the loading control. **(B)** TCR-dependent calcium release in P116 (ZAP-70 -/-) Jurkat T cells stably rescued with WT or C564S ZAP-70. Shown are representative single-cell responses measured by Fura-2 imaging. **(C)** Expression of CD69 T cell surface activation marker by P116 (ZAP-70 -/-) Jurkat T cells stably rescued with WT or C564S ZAP-70. Cells were stimulated for 24 h with plate-bound anti-CD3 antibody and analyzed by flow cytometry. Data shown are representative of 3 independent biological repeats and represented as mean ± SEM. **(D)** IL-2 production by P116 (ZAP-70 -/-) Jurkat T cells stably rescued with WT or acylation-deficient C564S ZAP-70. IL-2 concentrations were measured by ELISA in supernatants from resting cells or cells stimulated for 24 h with plate-bound anti-CD3 antibody. Data shown are representative of 3 independent biological repeats and represented as mean ± SEM.


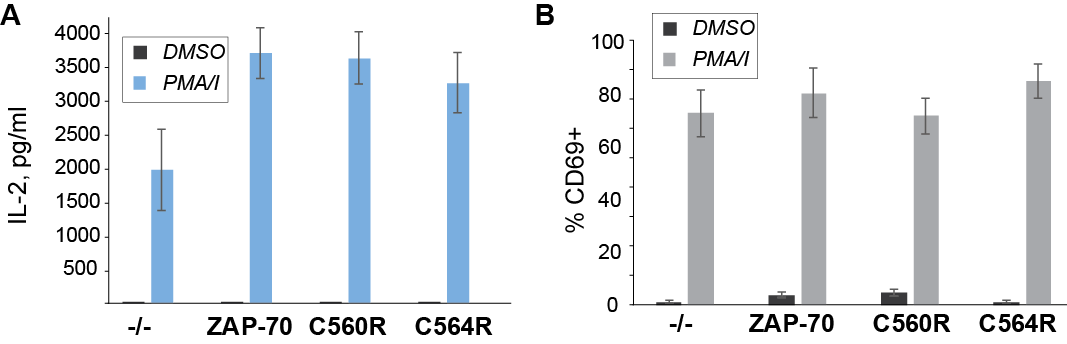


**Fig. S5. Signaling downstream of ZAP-70 is not affected in cells expressing C564R ZAP-70. (A)** IL-2 production by P116 (ZAP-70 -/-) Jurkat T cells stably rescued with ZAP-70 variants. IL-2 concentrations were measured by ELISA in supernatants from resting cells or cells stimulated for 6 h with PMA/Ionomycin. Data shown are representative of three independent experiments and represented as mean ± SEM. **(B)** Expression of CD69 T cell surface activation marker by P116 stably rescued with ZAP-70 variants. Cells were stimulated for 6 h with PMA/Ionomycin and analyzed by flow cytometry. Data shown are pooled from 3 independent experiments and represented as mean ± SEM.


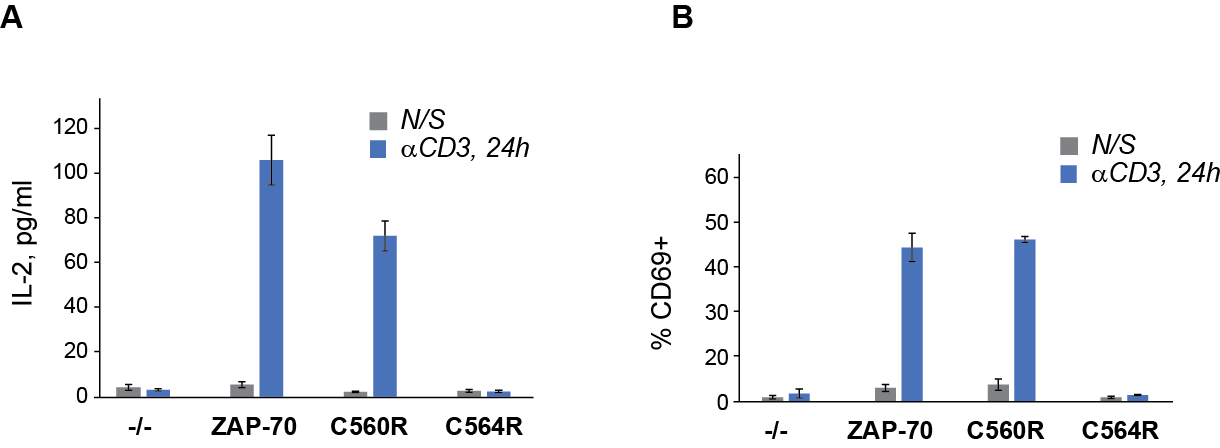


**Fig. S6. Cysteine to arginine substitution at the position 560 does not affect ZAP-70-mediated T cell activation. (A)** IL-2 production by P116 (ZAP-70 -/-) Jurkat T cells stably rescued with ZAP-70 variants. IL-2 concentrations were measured by ELISA in supernatants from resting cells or cells stimulated for 24 h with plate-bound anti-CD3 antibody. Data shown are representative of three independent experiments and represented as mean ± SEM. **(B)** Expression of CD69 T cell surface activation marker by P116 stably rescued with ZAP-70 variants. Cells were stimulated for 24 h with plate-bound anti-CD3 antibody and analyzed by flow cytometry. Data shown are representative of 3 independent biological repeats and represented as mean ± SEM.
